# Supplementary material for: Estimation of Protein and Amino Acid Requirements in Layer Chicks Depending on Dynamic Model
Source: Animals (Basel). 2024 Feb 29;14(5):764. doi: 10.3390/ani14050764 (PMC10930605; doi:10.3390/ani14050764)
Supplement: Supplementary file 1 [file animals-14-00764-s001.zip › animals-2861680-supplementary.pdf]

Table S1. Composition and nutritional values of the basal diet

item	Day 0-14	Day 15-42
Ingredient, %		
Corn	52.11	54.07
Soybean meal	34.00	31.35
Wheat bran	2.50	3.20
Soybean oil	4.00	4.00
$CaHPO_4$	0.50	0.46
$CaCO_3$	2.18	2.24
Salt	0.42	0.42
Lysine	0.07	0.03
Methionine	0.17	0.17
Cystine	0.05	0.04
Threonine	-	0.02
Vitamin and mineral premix[1]	4	4
Total	100	100
Nutrient composition[2]		
ME, MJ/kg	2.88	2.89
Crude protein, %	19.92	19.02
Lysine, %	1.10	1.01
Methionine, %	0.46	0.45
Methionine+Cysteine, %	0.84	0.81
Threonine, %	0.75	0.73
Na, %	0.18	0.18
Cl, %	0.30	0.30
Ca, %	1.00	1.00
Available Phosphorus, %	0.46	0.45

[1]Vitamin and mineral premix provided/kg diet: iron, 100 mg; copper, 8 mg; manganese, 20 mg; zinc, 100 mg; selenium, 0.3 mg; iodine, 0.7 mg; retinyl acetate, 10280 IU; cholecalciferol 2280 IU; dl-a-tocopheryl acetate,17.12 mg; menadione, 6.82 mg; thiamin, 2.28 mg; riboflavin, 5.68 mg; pantothenic acid, 12.25 mg; pyridoxine, 2.28 mg; niacin, 22.84 mg; biotin,0.18 mg; folic acid, 1.12 mg.

[2]Caculated.

Table S2. Composition and nutritional values of the nitrogen-free diet and low-nitrogen diet

Item	Nitrogen-free	Low-nitrogen
Ingrediant(%)		
Corn starch	67.50	57.50
Glucose	19.90	22.9
Cellulose	3.00	4.40
Soybean oil	1.60	1.60
CaHPO4	2.65	2.60
CaCO3	0.90	0.90
Casein	-	5.65
Salt	0.45	0.45
Vitamin and mineral premix[1]	4	4
Total	100	100
Nutrient composition[2]		
ME, MJ/kg	2.88	2.89
Crude protein, %	0.26	5.01
Lysine, %	-	
Methionine, %	-	0.15
Methionine+Cysteine, %	-	0.17
Threonine, %	-	0.21
Na, %	0.18	0.18
Cl, %	0.27	0.28
Ca, %	0.90	0.91
Available Phosphorus, %	0.46	0.46

[1]Vitamin and mineral premix provided/kg diet: iron, 100 mg; copper, 8 mg; manganese, 20 mg; zinc, 100 mg; selenium, 0.3 mg; iodine, 0.7 mg; retinyl acetate, 10280 IU; cholecalciferol 2280 IU; dl-a-tocopheryl acetate,17.12 mg; menadione, 6.82 mg; thiamin, 2.28 mg; riboflavin, 5.68 mg; pantothenic acid, 12.25 mg; pyridoxine, 2.28 mg; niacin, 22.84 mg; biotin,0.18 mg; folic acid, 1.12 mg.

[2]Caculated.

Table S3. Live weight and corresponding body composition of Jing Tint 6 chicks at different days of age [1]

Days of age	0	7	14	21	28	35	42
Body weight, g	37.65±0.02	56.77±0.71	96.63±0.91	145.93±0.99	202.52±1.17	287.83±5.14	391.82±6.58
Carcass/body weight, %	96.18±0.10	96.37±0.09	96.96±0.11	97.04±0.07	96.34±0.13	95.78±0.12	93.51±0.29
Feather/body weight, %	3.82±0.10	3.63±0.09	3.04±0.11	2.96±0.07	3.66±0.14	4.22±0.12	6.49±0.30
Carcass protein content, %	15.18±0.19	15.00±0.13	16.61±0.34	18.17±0.14	18.30±0.24	18.42±0.22	17.77±0.26
Feather protein content, %	87.79±0.46	87.89±0.62	88.72±0.57	87.10±0.59	87.06±0.65	86.55±0.50	85.75±0.95
Total carcass protein, g	4.80±0.11	7.11±0.21	15.14±0.51	25.66±0.22	36.26±0.48	48.11±0.71	64.19±1.89
Total feather protein, g	1.26±0.03	1.81±0.04	2.62±0.11	3.76±0.10	6.46±0.26	10.49±0.31	21.90±1.22

[1] Values are means ± SE, n = 6.

Table S4. Amino acid pattern of protein (% of protein) in de-feathered carcasses of Jing Tint 6 chicks [1]

Days of Age	Aspartic acid	Threonine	Serine	Glutamic acid	Glycine	Alanine	Valine	Cystine	Methionine	Isoleucine	Leucine	Tyrosine	Phenylalanine	Histidine	Lysine	Arginine	Proline	Tryptophan
0	7.25±0.07^{a}	4.36±0.05^{a}	4.90±0.15^{a}	13.23±0.43^{a}	7.65±0.09^{a}	7.92±0.13^{a}	4.25±0.12^{a}	1.97±0.25^{a}	2.65±0.08^{ab}	3.70±0.07^{ab}	6.62±0.17^{a}	2.59±0.06^{b}	3.88±0.08^{c}	2.30±0.06^{ab}	5.04±0.06^{b}	5.31±0.06^{ab}	5.61±0.13^{a}	0.90±0.03^{c}
7	7.69±0.32^{ab}	3.55±0.23^{ab}	3.42±0.35^{b}	11.48±0.59^{b}	7.25±0.51^{a}	6.77±0.45^{b}	4.32±0.22^{a}	1.90±0.15^{a}	2.61±0.06^{ab}	3.87±0.14^{b}	6.87±0.35^{a}	2.87±0.14^{c}	3.88±0.15^{c}	1.90±0.17^{a}	5.64±0.31^{a}	5.69±0.42^{a}	5.62±0.30^{a}	0.72±0.03^{b}
14	7.01±0.12^{b}	3.90±0.29^{ab}	4.15±0.39^{ab}	11.74±0 .36^{b}	7.01±0.42^{a}	7.46±0.50^{ab}	4.00±0.09^{a}	2.14±0.25^{a}	2.65±0.05^{ab}	3.55±0.08^{ac}	6.33±0.21^{b}	2.56±0.05^{b}	3.64±0.09^{ac}	2.35±0.20^{b}	5.28±0.11^{ab}	5.26±0.22^{ab}	5.69±0.20^{a}	0.58±0.07^{a}
21	6.25±0.05^{c}	3.66±0.05^{ab}	3.51±0.10^{b}	11.71±0.21^{b}	7.10±0.08^{a}	7.00±0.08^{ab}	4.37±0.09^{a}	1.86±0.16^{a}	2.51±0.12^{b}	3.51±0.04^{ac}	6.18±0.06^{b}	2.03±0.03^{a}	3.45±0.05^{ab}	2.88±0.04^{c}	4.92±0.06^{bc}	5.04±0.13^{b}	4.15±0.05^{b}	0.53±0.03^{a}
28	6.10±0.11^{c}	3.72±0.15^{b}	3.53±0.14^{b}	11.45±0 .21^{b}	7.38±0.16^{a}	7.28±0.24^{ab}	3.42±0.17^{b}	1.56±0.15^{a}	2.63±0.06^{ab}	3.41±0.04^{c}	5.75±0.19^{b}	2.03±0.05^{a}	3.36±0.06^{ab}	3.02±0.11^{c}	4.56±0.06^{c}	4.80±0.16^{b}	4.59±0.16^{b}	0.70±0.03^{b}
35	6.04±0.13^{c}	3.63±0.09^{ab}	3.49±0.15^{b}	11.35±0.24^{b}	6.80±0.11^{a}	6.74±0.24^{b}	2.75±0.33^{c}	1.85±0.20^{a}	2.48±0.06^{b}	3.54±0.07^{ac}	6.24±0.19^{b}	2.05±0.10^{a}	3.43±0.09^{ab}	2.92±0.03^{c}	5.05±0.09^{b}	4.68±0.04^{b}	4.22±0.12^{b}	0.72±0.01^{b}
42	5.94±0.08^{c}	3.42±0.10^{b}	3.58±0.10^{b}	11.21±0.4^{b}	6.92±0.28^{a}	6.67±0.26^{b}	3.70±0.12^{c}	2.22±0.34^{a}	2.78±0.06^{a}	3.41±0.04^{c}	5.81±0.09^{b}	2.10±0.06^{a}	3.19±0.11^{b}	2.84±0.07^{c}	4.81±0.17^{bc}	4.88±0.16^{b}	4.23±0.08^{b}	0.57±0.03^{a}
Average of 0-2 week	7.32±0.20	4.01±0.11	4.31±0.21	12.42±0.24	7.23±0.12	7.63±0.11	4.17±0.12	2.01±0.12	2.63±0.04	3.71±0.09	6.61±0.16	2.67±0.10	3.80±0.08	2.23±0.07	5.32±0.17	5.42±0.13	5.65±0.04	0.73±0.04
Average of 3-6 week	6.08±0.06	3.52±0.07	3.47±0.05	11.29±0.15	6.84±0.09	6.78±0.10	3.26±0.30	1.87±0.12	2.60±0.04	3.46±0.04	6.01±0.12	2.14±0.06	3.36±0.06	2.77±0.08	4.84±0.09	4.75±0.10	4.38±0.15	0.63±0.02

[1] Values are means ± SE, n = 6. Means in a row with different superscripts are different, $P < 0.05$.

Table S5. Amino acid pattern of protein (% of protein) in feather of Jing Tint 6 chicks [1]

Days of Age	Aspartic acid	Threonine	Serine	Glutamic acid	Glycine	Alanine	Valine	Cystine	Methionine	Isoleucine	Leucine	Tyrosine	Phenylalanine	Histidine	Lysine	Arginine	Proline	Tryptophan
0	4.02±0.23^{c}	2.35±0.13^{c}	4.96±0.35^{c}	5.40±0.30^{c}	3.26±0.26^{c}	2.26±0.22^{a}	4.25±0.26^{d}	6.11±0.31^{a}	0.85±0.08^{a}	2.61±0.16^{a}	4.25±0.24^{a}	2.42±0.16^{ab}	2.93±0.14^{c}	0.65±0.08^{a}	1.18±0.14^{a}	3.94±0.49^{a}	7.45±0.06^{ab}	0.60±0.03^{a}
7	5.69±0.19^{ab}	3.35±0.12^{a}	7.69±0.17^{ab}	8.64±0.17^{ab}	5.42±0.23^{ab}	4.17±0.27^{b}	5.28±0.18^{ab}	6.80±0.30^{abc}	0.81±0.05^{a}	3.38±0.15^{b}	5.58±0.22^{b}	2.76±0.17^{a}	3.80±0.14^{a}	0.98±0.09^{b}	1.40±0.08^{ab}	5.24±0.34^{b}	8.33±0.33^{ab}	0.46±0.03^{b}
14	6.08±0.20^{b}	3.59±0.10^{ab}	7.59±0.29^{ab}	8.60±0.26^{ab}	5.77±0.19^{a}	4.16±0.48^{b}	5.58±0.19^{abc}	6.85±0.23^{abc}	0.87±0.05^{a}	3.30±0.10^{b}	5.63±0.19^{b}	1.99±0.08^{b}	3.59±0.13^{ab}	0.89±0.05^{b}	1.70±0.07^{cd}	5.59±0.21^{b}	8.17±0.29^{ab}	0.37±0.02^{c}
21	5.42±0.14^{a}	3.32±0.10^{a}	7.45±0.26^{a}	9.19±0.23^{a}	4.93±0.15^{b}	4.93±0.15^{b}	5.07±0.14^{a}	6.74±0.20^{abc}	0.86±0.03^{a}	3.48±0.17^{b}	5.34±0.15^{b}	2.10±0.08^{b}	3.38±0.09^{b}	0.63±0.03^{a}	1.54±0.08^{bc}	4.58±0.14^{ab}	7.94±0.17^{ab}	0.31±0.02^{c}
28	3.20±0.11^{d}	1.98±0.10^{d}	4.19±0.30^{d}	4.92±0.48^{c}	2.88±0.43^{c}	2.73±0.65^{a}	3.86±0.20^{d}	6.60±0.35 ab	0.86±0.04^{a}	2.65±0.32^{a}	3.95±0.30^{a}	2.33±0.21^{ab}	2.46±0.17^{d}	0.22±0.02^{c}	0.91±0.05^{e}	3.68±0.69^{a}	7.97±0.60^{ab}	0.30±0.01^{c}
35	5.80±0.27^{ab}	3.61±0.11^{ab}	7.24±0.16^{a}	8.28±0.16^{b}	5.20±0.07^{ab}	5.07±0.16^{b}	5.73±0.14^{bc}	7.67±0.35^{c}	0.89±0.05^{a}	3.64±0.04^{b}	6.00±0.05^{b}	2.61±0.11^{a}	3.64±0.02^{ab}	0.62±0.01^{a}	1.82±0.04^{d}	5.28±0.16^{b}	8.03±0.22^{ab}	0.31±0.02^{c}
42	5.87±0.08^{ab}	3.84±0.06^{b}	8.33±0.12^{b}	9.08±0.21^{ab}	5.62±0.12^{ab}	4.43±0.10^{b}	5.95±0.11^{c}	7.49±0.30^{bc}	0.77±0.03^{a}	3.58±0.06^{b}	5.84±0.13^{b}	1.99±0.08^{b}	3.60±0.07^{ab}	0.51±0.02^{a}	1.49±0.05^{bc}	5.42±0.18^{b}	8.85±0.32^{b}	0.21±0.02^{d}
Average of 0-2 week	5.89±0.20	3.47±0.12	7.64±0.05	8.63±0.02	5.59±0.18	4.16±0.01	5.43±0.15	6.82±0.19	0.84±0.03	3.34±0.04	5.60±0.03	2.37±0.38	3.69±0.11	0.93±0.05	1.55±0.15	5.42±0.18	8.25±0.08	0.42±0.09
Average of 3-6 week	5.70±0.20	3.59±0.22	7.67±0.47	8.85±0.41	5.25±0.28	4.40±0.56	5.58±0.37	7.29±0.25	0.81±0.03	3.57±0.06	5.73±0.28	2.19±0.35	3.54±0.11	0.58±0.05	1.62±0.15	5.09±0.37	8.27±0.41	0.27±0.07

[1] Values are means ± SE, n = 6. Means in a row with different superscripts are different, $P < 0.05$.
